# Evaluating human perceptions of android robot facial expressions based on variations in instruction styles

**DOI:** 10.3389/frobt.2025.1728647

**Published:** 2025-12-16

**Authors:** Ayaka Fujii, Carlos Toshinori Ishi, Kurima Sakai, Tomo Funayama, Ritsuko Iwai, Yusuke Takahashi, Takatsune Kumada, Takashi Minato

**Affiliations:** 1 Guardian Robot Project, RIKEN, Kyoto, Japan; 2 Hiroshi Ishiguro Laboratories, Advanced Telecommunications Research Institute International, Kyoto, Japan; 3 Graduate School of Education, Kyoto University, Kyoto, Japan; 4 Graduate School of Informatics, Kyoto University, Kyoto, Japan

**Keywords:** human-robot interaction, facial expression, android robot, emotion attribution, social agency

## Abstract

Robots that interact with humans are required to express emotions in ways that are appropriate to the context. While most prior research has focused primarily on basic emotions, real-life interactions demand more nuanced expressions. In this study, we extended the expressive capabilities of the android robot Nikola by implementing 63 facial expressions, covering not only complex emotions and physical conditions, but also differences in intensity. At Expo 2025 in Japan, more than 600 participants interacted with Nikola by describing situations in which they wanted the robot to perform facial expressions. The robot inferred emotions using a large language model and performed corresponding facial expressions. Questionnaire responses revealed that participants rated the robot’s behavior as more appropriate and emotionally expressive when their instructions were abstract, compared to when they explicitly included emotions or physical states. This suggests that abstract instructions enhance perceived agency in the robot. We also investigated and discussed how impressions towards the robot varied depending on the expressions it performed and the personality traits of participants. This study contributes to the research field of human–robot interaction by demonstrating how adaptive facial expressions, in association with instruction styles, are linked to shaping human perceptions of social robots.

## 1 Introduction

Robots that interact with humans are required to express emotions appropriately according to the situation. Facial expressions are known to have a major impact on expressing emotions in human-robot interaction (Sato et al., 2025). Many studies have shown that facial expressions can enhance the warmth and attractiveness of the robot and affect the trust and impressions towards the robot (Ghazali et al., 2018; Mishra et al., 2023; Dong et al., 2023).

In addition to a robot’s ability to express emotions through facial expressions, how humans attribute emotions to the robot is also an important factor in realizing rich emotional interactions. Most previous research on emotion attribution has primarily relied on an approach in which researchers intentionally vary robot behaviors or experimental conditions (Thellman et al., 2022), while the influence of human behavior on how robots are perceived has received much less attention. In particular, it is still unclear how different styles of instruction to a robot influence the emotion attribution to the robot. A previous study on end-user programming shows that when users customize a robot’s behavior, using a more abstract interface leads to a higher perceived social agency of the robot, compared to using a detailed interface (Zhang et al., 2025). This result implies the effect of abstract instructions in prompting users to derive interpretations of the robot’s process of inference.

In this study, we hypothesize that such a mechanism could extend to emotional contexts. Based on the hypothesis, we design an experiment in which participants provide situational instructions to the robot to perform facial expressions, and investigate whether the abstractness of the instructions influences their emotion attribution to the robot. We assume that when participants provide abstract instructions without emotions for specifying the context for performing facial expressions, they may build a mental model of the robot’s internal state based on predictive processing (Clark, 2013). This inference process can involve not only cognitive capacities (Agency), but also emotional capacities (Experience) of the robot (Gray et al., 2007). Accordingly, we examine the hypothesis that perceived agency and experience of the robot increase when participants specify only abstract situations rather than explicitly including emotions or physical conditions.

In order to conduct the experiment, a robot capable of performing a wide range of facial expressions in response to various situations is essential. Research on robotic facial expression generation has traditionally focused on the expression of basic emotions, such as happiness, anger, sadness, fear, surprise, and disgust (Kobayashi and Hara, 1993; Allison et al., 2008; Faraj et al., 2021; Sato et al., 2022). However, in real-life settings, people often expect not only basic emotions such as “angry” or “sad,” but also more nuanced expressions like “noticing failure” or “drowsy.” In addition to the range of capable expressions, the ability to select appropriate expressions according to the situation is also important.

To meet these requirements, we select an android robot named Nikola (Sato et al., 2022), which is one of the robots equipped with a large number of facial actuators, allowing for the detailed expression of subtle emotional nuances. Prior research indicates that embodiment and physical presence contribute to improving recognition of specific facial expressions in agents (Mollahosseini et al., 2018). Therefore, we adopt a physically embodied robot capable of expressing a wide range of facial expressions, rather than a virtual agent, to sufficiently investigate how people attribute emotions to robots. In addition, Nikola is designed to resemble a child so that it can interact naturally with both adults and children. As we conduct the large-scale experiment at Expo 2025 in Japan, which involves participants across a wide range of age groups, Nikola serves as an ideal platform for the experiment. In the previous study, our research team has demonstrated that Nikola can express around twenty complex emotions, such as boredom and hesitation (Diel et al., 2025). In this study, we extend Nikola to be capable of displaying over 60 types of facial expressions, and investigate how participants evaluated the expressions performed by the robot in response to their instructions about the situations.

The contributions of this study are 2-fold:

1. We evaluate the effect of the abstractness of instructions to the robot on emotion attribution. We conduct a large-scale experiment outside the laboratory setting, enabling the collection of data on more natural human reactions.
2. We implement 63 facial expressions, including complex emotions and physical conditions, in an android robot, and verify whether the robot can appropriately perform the facial expressions in response to user-specified situations.

## 2 Related works

### 2.1 Variety of facial expressions

Many studies have been conducted on the classification of human facial expressions. Ekman’s six basic emotions (happiness, anger, sadness, fear, surprise, and disgust) (Ekman and Friesen, 1971) are widely known and have been referenced in many robot research (Kobayashi and Hara, 1993; Allison et al., 2008; Faraj et al., 2021; Sato et al., 2022). However, recent research on human facial expressions has shown that the repertoire of facial expressions is not limited to the basic expressions. Du et al. studied compound facial expressions that combine different emotions and defined 21 different emotion categories (Du et al., 2014). For example, happily surprised and angrily surprised are different compound emotion categories. Cowen et al. showed that facial and body expressions can be used to express at least 28 different categories of emotions, including complex emotions such as embarrassment and relief (Cowen and Keltner, 2020).

It is also known that facial expressions reflect not only emotions but also physical conditions such as fatigue and pain. For example, Sundelin et al. showed that sleep deprivation affects facial characteristics related to the eyes, mouth, and skin (Sundelin et al., 2013). Numerous studies have also examined the use of facial expressions to assess pain (Prkachin, 2009; Werner et al., 2017). Furthermore, Strack et al. found that facial expressions when looking at food differ between when hungry and when full (Strack et al., 2009).

In addition, many studies show that the intensity of emotions changes the way facial expressions are made. Ekman et al. showed that the frequency and intensity of specific facial expressions change depending on the intensity of negative emotions (Ekman et al., 1980). Kunz et al. found a correlation between the degree of subjective pain and changes in facial expressions (Kunz et al., 2004).

However, in robotics research, facial expression generation has traditionally been based on basic emotion models, such as Ekman’s six categories. While this approach enables standardized evaluation, it limits the diversity and contextual flexibility of expressions. To address this limitation, we expand the expressive capabilities of the android robot, Nikola, by implementing 63 distinct facial expressions, including variations in intensity. We incorporate not only basic emotions, but also complex emotions and expressions reflecting physical conditions to realize richer emotional interactions.

### 2.2 Emotion interpretation with context

An important aspect of emotional interaction is not only whether a robot can display easy-to-identify facial expressions, but also whether humans can feel emotions from the robot in a given context. Psychological studies have shown that emotion perception from facial expression is highly context-dependent. Carroll et al. demonstrated that facial expressions can be interpreted as different emotions depending on the context, rather than being interpreted as the emotion when seen in isolation (Carroll and Russell, 1996). For example, an expression that appears to be anger on its own tends to be interpreted as fear in a context of fear, and an expression that appears to be sadness on its own tends to be interpreted as disgust in a context of disgust. There are also numerous reports that neutral facial expression can be interpreted as different emotion expressions depending on the context (Barratt et al., 2016; Calbi et al., 2017).

In the case of robots as well, it is also known that facial expressions are recognized differently depending on contextual information (Bennett and abanović, 2015), though some studies have shown that facial expressions that deviate from the context can negatively impact the trust and impression towards the robot (Paradeda et al., 2016; Appel et al., 2021). These findings suggest that, rather than attempting to produce one correct facial expression, it would be more reasonable to design a system that generates expressions within a range perceived as appropriate by humans, depending on the situational context.

However, most previous studies on robotic facial expressions have followed a paradigm in which the robot first displays an expression, and then human participants are asked to identify which emotion it represents. In our prior study using Nikola, where various complex expressions were displayed without contextual information, some expressions tended to be judged to reflect emotions other than their intended targets (Diel et al., 2025). Though the result suggests that the expression is also perceived as appropriate in other contexts, it does not imply that it is inappropriate in the context of the target emotion.

Despite the importance of context in interpreting facial expressions, little attention has been paid to whether a robot’s expression is perceived as contextually appropriate, particularly when the context is imagined by the user. To address this gap, this study adopts a context-first approach: participants imagine and specify a situation at first, and the robot then displays a facial expression that corresponds to the situation. This enables the evaluation of the contextual appropriateness and emotion interpretation of facial expressions.

### 2.3 Emotion attribution

Agency and Experience are key dimensions of mind perception (Gray et al., 2007). Agency refers to the capacity to think and act, such as self-control, planning, reasoning, and decision-making, whereas Experience refers to the capacity to feel sensations and emotions, such as pain, hunger, pleasure, fear, and embarrassment. In the context of human-robot interaction, the extent to which these factors are attributed to a robot has been shown to influence trust, anthropomorphism, and satisfaction (Yam et al., 2021; Spatola and Wudarczyk, 2021; Esterwood and Robert, 2023).

Previous research on emotional attribution toward robots has largely examined factors such as robotic behavior and appearance, as well as the influence of human factors such as age and social background (Haring et al., 2015; Abubshait and Wiese, 2017; Manzi et al., 2020). However, little attention has been paid to how humans’ behavior, such as the way they interact with or give instructions to a robot, including the degree of abstraction in the instructions, affects their perception of the robot. To investigate this underexplored issue, we examine whether emotion attribution to the robot changes according to the way of giving instructions to the robot.

## 3 Materials and methods

### 3.1 Hypotheses

Research on how humans give instructions affects emotion attribution to robots remains underdeveloped. In the field of end-user programming, research shows that customizing the behavior of home assist robots through low-granularity interfaces enhances perceived social agency compared to high-granularity interfaces (Zhang et al., 2025). This perceived social agency includes autonomy, perceived agency, social presence, social intelligence, and perceived intelligence. It is suggested that the abstractness of user instructions may influence the emotion attribution to the robot. Similarly, in the task of performing facial expressions, which is an important factor in human-robot interaction, we predict that abstract instructions can enhance emotion attribution, as the robot is more likely to be perceived as inferring the appropriate emotion on its own. However, this prediction has not yet been empirically tested because of the technical limitations of conventional robots, which lack the ability to perform nuanced emotional expressions in response to diverse user instructions.

In this study, we utilize the android robot, Nikola, which is capable of performing over 60 types of facial expressions, in order to investigate the prediction. Leveraging Nikola’s rich expressive capabilities, we introduce a novel interactive framework in which participants freely describe a situation, and the robot performs a facial expression appropriate to that situation. This setup enables testing the effect of instructional style on emotion attribution. We examine whether emotion attribution to the robot changes when participants specify only abstract situations, compared to when their instructions include specific emotions or physical conditions, based on the following three hypotheses.

H1: Abstract instructions lead to higher perceptions of the robot giving appropriate responses (Agency).

H2: Abstract instructions lead to higher perceptions of the robot expressing emotions (Agency).

H3: Abstract instructions lead to higher perceptions of the robot showing empathy (Experience).

### 3.2 Overview of the experiment

**FIGURE 1:**
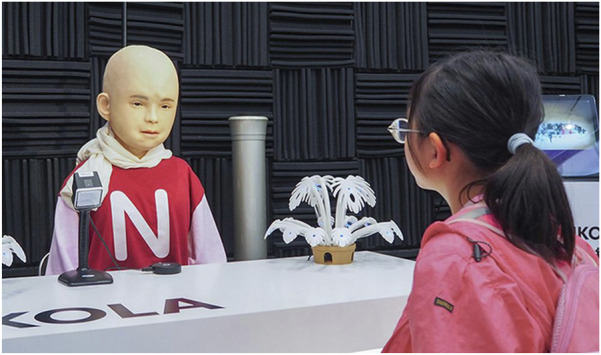
Experiment scene at the Expo 2025 Osaka, Kansai, Japan.

We conducted a 7-day experiment at the Expo 2025 Osaka, Kansai, Japan, from May 20 to 26 May 2025. Nikola (Sato et al., 2022), which is an android robot capable of performing facial expressions, was employed in the study. In each interaction, the participant specified scenes or situations to the robot, and the robot inferred the emotion and performed the corresponding facial expression. An example of the experiment scene is shown in Figure 1. We also performed an exploratory analysis of the relationship between personality traits based on the Big Five (Iwai et al., 2019) and impressions towards the robot.

### 3.3 Android robot system

Nikola is an android robot with the appearance of a junior high school-aged child. It is capable of performing various facial expressions and head movements. Previous experiments have demonstrated that it can express not only the basic emotions (Yang et al., 2022) but also more complex expressions (Diel et al., 2025). There are 35 low-noise pneumatic actuators: 29 dedicated to facial movements, 3 for eye movements (left eye pan, right eye pan, and tilt motion of both eyes), and 3 for neck movements (roll, pitch, and yaw). The frontal facial surface and neck area are covered with soft silicone skin.

The demonstration system is implemented using Robot Operating System (ROS), integrating human tracker, audio tracker, speech recognition, and speech synthesis functionalities. These core modules are built upon the interaction system described in previous research (Glas et al., 2016). The human tracker is based on distance measurement using a stereo camera to estimate human positions near the robot. The audio tracker detects the speech activities of humans near the robot, based on sound source localization using two microphone arrays (Ishi et al., 2015). The speech signal of the target user who is interacting with the robot is then separated from interfering sound sources (Ishi et al., 2018), and sent to the speech recognition module.

### 3.4 Implementation of facial expression

**FIGURE 2:**
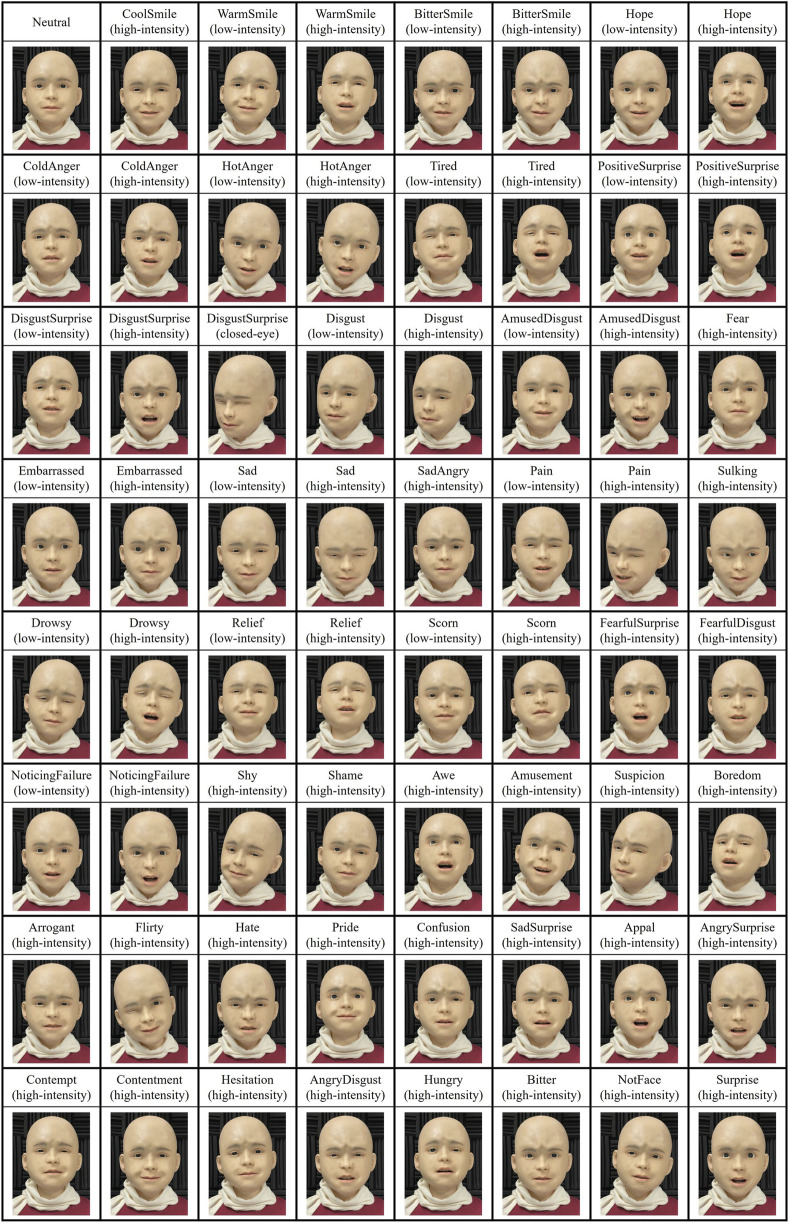
Nikola’s neutral face and 63 facial expressions prepared for this experiment.

Based on prior research on facial expressions (McDaniel et al., 2007; Vural et al., 2007; Krumhuber et al., 2013; Du et al., 2014; Benitez-Quiroz et al., 2016; Cordaro et al., 2018; Barrett et al., 2019), we prepared 63 facial expressions, as shown in Figure 2. These facial expressions were selected considering their relevance in situations specified during the test trials before the experiment and their typical appearance in daily life. They included not only basic emotions but also complex emotions and expressions reflecting physical conditions. Each expression was assigned one to four emotion labels that matched its characteristics, resulting in a total of 127 labels. For example, BitterSmile (high intensity) was labeled as expressing a derisive and mocking smile, while NotFace (high intensity) was labeled as disagreement.

To validate these facial expressions, we conducted an online survey using static photos of these expressions with a total of 121 participants. On average, 4.79 participants (ranging from 4 to 7) evaluated each expression. For each expression, we calculated the percentage of participants who judged it as appropriate, and the average of these percentages across all expressions was 82.5%. Sixty-two expressions were judged as appropriate by at least 50% of the participants, and one expression (Hungry) was judged appropriate by 40% of participants. These results suggest that the implemented facial expressions were generally perceived as appropriate, though some expressions showed variability in interpretation across participants, indicating potential limitations in their generalizability as facial expressions.

Using GPT-4o by OpenAI, Nikola inferred the emotion labels that closely matched the sentiment associated with a participant-specified situation. The described situation, along with all emotion labels, was input into the prompt, which also included an instruction for the model to output three emotion labels corresponding to the given situation. Subsequently, it showed the facial expressions corresponding to one of the inferred emotion labels.

### 3.5 Design of dialogue interaction flow

In the introduction phase, the robot first introduced itself and explained that it was skilled at imagining human emotions and expressing them through facial expressions. At first, the robot presented an example situation and performed the facial expression corresponding to that scene. For example, the robot said “when I received a large pocket money, but found out that my friend received even more” and performed the facial expression of NotFace (high-intensity). This initial example and its corresponding facial expression were randomly selected from a set of three pre-prepared options.

The facial expression trial phase was conducted twice in total. In each phase, the robot first asked the participant to describe a situation from their past experiences in which they would like the robot to perform a facial expression. Participants freely spoke about situations that came to mind, and no specific guidance was given regarding the level of abstraction of the speech. The participant’s speech was transcribed using an automatic speech recognition system implemented through the Web Speech API provided by Google Chrome. The transcribed content was then summarized using GPT-4o. The robot verbally confirmed the summary with the participant. If the participant did not disagree, the robot proceeded to perform a facial expression. In the case in which the participant’s speech was not detected within the time limit, the robot suggested a situation from the pre-prepared list and then proceeded to perform a facial expression. GPT was prompted to select three suitable emotion labels from the entire label set. Using one of these labels, the robot performed a corresponding facial expression. After the facial expression, the robot asked the participant for their impression of it. If the participant responded positively, the interaction moved to the next phase. If the participant responded negatively, the robot performed an alternative expression corresponding to a different inferred emotion label. If all three inferred emotions received negative responses, the phase concluded, and the interaction moved on. In the second trial phase, the robot asked the participant to provide a different situation, and the subsequent interaction followed the same process as the first trial phase.

After the second trial phase, the closing phase began. Based on the participant’s reaction history to the performed expressions, the robot provided a brief comment on the interaction. Finally, the robot said a farewell message, and the demonstration ended.

### 3.6 Experiment procedure

Informed consent was obtained from all participants before the experiment. Before interacting with the robot, participants completed a questionnaire about their background, including age, gender, and Big Five personality traits. The interaction demonstration with the robot lasted approximately 4 min in total.

Following the demonstration, participants completed a post-experience questionnaire. The questionnaire consisted of three items (Q1–Q3), each rated on a four-point Likert scale, where 1 indicated “Not at all” and 4 indicated “Very much.” The scale excluded a neutral midpoint intentionally.

Q1: The robot responded appropriately to what I said.

Q2: The robot expressed emotions towards me.

Q3: The robot empathized with my feelings.

These three items were extracted from Iwai et al. (2025) to align with the aim of this experiment and corresponded to the hypotheses H1–H3. Three of the authors created a child-appropriate version of the pre- and post-experiment questionnaires for participants aged 6–15, using child-friendly expressions that conveyed the same meaning. In addition, participants aged 16 and over rated their familiarity with each of seven types of robot-related technologies, including cleaning robots, serving robots, guide robots, smart speakers, pet robots, companion robots, and care robots, using a 5-point Likert scale. A rating of 1 indicated that the participant did not know the technology, while a rating of 5 indicated that they frequently used (or had used) it.

The experiment was conducted with the approval of the ethics committee of RIKEN (Permission number: Wako2025-31). It was audio-recorded, and participants’ utterances were analyzed by combining the results of speech recognition with transcripts produced after the experiment.

### 3.7 Participants

The participants were visitors to the Expo 2025. The number of visitors was not controlled, and only individuals who had given their consent were included in the study. Individuals who did not provide consent only experienced the interaction, without participating in the research.

After excluding cases with major system errors, there were 624 people with corresponding data from the robot interaction log and questionnaires. 252 were male, 368 were female, and 4 identified as other. Ages ranged from 6 to 86 years, with a mean age of 48.6 years. All participants were able to understand Japanese, since the interaction and questionnaire were conducted in Japanese. Participants aged between 6 and 17 years were included in the study only if consent was obtained from their accompanying guardian. Young children participated in the interaction and completed the questionnaire with the assistance of accompanying adults.

## 4 Results

### 4.1 Interaction system evaluation

#### 4.1.1 Unsmooth interaction flow

Since 624 participants completed two facial expression trials each, a total of 1,248 trials were conducted. There were 177 cases (14.2%) in which the robot was unable to recognize speech from the participant within the time limit, and proposed a situation to proceed with the interaction. The reasons for these cases include failures in speech recognition and the inability of the participants to come up with a situation. We compared participants who experienced at least one case in which the robot proposed a situation (n = 155) with those who did not (n = 469). The median (mean ± SE) scores of the former group vs. the latter group were as follows: Q1: 3.00 (2.93 ± 0.0648) vs. 4.00 (3.51 ± 0.0309), Q2: 3.00 (3.11 ± 0.0631) vs. 4.00 (3.43 ± 0.0322), Q3: 3.00 (2.87 ± 0.0605) vs. 3.00 (3.26 ± 0.0349). The one-sided Mann–Whitney U test showed that the former group gave significantly lower questionnaire ratings across all items (Q1: U = 50988.0, p < 0.001, Q2: U = 44822.5, p < 0.001, Q3: U = 46493.5, p < 0.001).

There were a total of 68 cases (5.4%) where the robot’s summary of the speech recognition results contained obvious errors. The causes of errors include cases where GPT-4o determined that there was insufficient information and did not summarize, and cases where the speech recognition ended midway through the participants’ speech, resulting in the opposite meaning of the summary. When comparing participants who experienced at least one summary error (n = 67) with those who did not (n = 557), the median (mean ± SE) scores of the former group vs. the latter group were as follows: Q1: 3.00 (2.91 ± 0.0844) vs. 4.00 (3.42 ± 0.0312), Q2: 3.00 (3.21 ± 0.0940) vs. 3.00 (3.37 ± 0.0308), Q3: 3.00 (3.03 ± 0.0876) vs. 3.00 (3.18 ± 0.0330). The former had significantly lower questionnaire ratings (Q1: U = 25923.0, p < 0.001, Q2: U = 20880.5, p = 0.039, Q3: U = 21034.0, p = 0.032).

In terms of facial expressions, the robot performed a different emotion once in 121 cases (9.6%) and twice in 43 cases (3.4%). Since a single trial could include up to three expressions, the maximum number of facial expression redos per trial was two. Some factors included the inability of the robot to identify an appropriate expression and unsuccessful summarization in the preceding stage, which in turn led to inappropriate expressions. Comparing participants who experienced at least one redo (n = 151) with those who did not (n = 473), the median (mean ± SE) scores of the former group vs. the latter group were as follows: Q1: 3.00 (3.18 ± 0.0580) vs. 4.00 (3.42 ± 0.0345), Q2: 3.00 (3.15 ± 0.0597) vs. 4.00 (3.42 ± 0.0331), Q3: 3.00 (2.98 ± 0.0612) vs. 3.00 (3.22 ± 0.0355). A comparison revealed that the former group gave significantly lower questionnaire ratings (Q1: U = 43073.0, p < 0.001, Q2: U = 43426.0, p < 0.001, Q3: U = 42121.0, p < 0.001).

#### 4.1.2 Analysis target: participants with smooth interaction flow

Based on the above results, the unsmooth interaction flows were considered to have influenced the evaluation of the experience. In order to investigate the effects of the instruction styles, we excluded unsmooth flows from subsequent analyses. The following analyses were then conducted with participants who experienced interactions with smooth flow (327 participants: 132 males, 194 females, and 1 other).

We compared participants included in the analysis (n = 327) and those excluded (n = 297) with respect to age group and familiarity with technology to examine the potential for selection bias due to the exclusion. For age, we classified participants into three categories: children ( ≤ 15 years, based on the use of child-friendly expressions in the questionnaire), older adults ( ≥ 65 years, based on the standard definition of older adults in Japan), and others (16–64 years). Among the included participants, there were 21 children, 46 older adults, and 260 others. Among the excluded participants, there were 19 children, 56 older adults, and 222 others. A chi-square test revealed no significant difference in age distribution between these two groups ( $\chi^{2}$ (2) = 2.64, p = 0.267). In addition, to compare the familiarity with technology, we analyzed the mean ratings across seven robot-related technologies. The included participants showed a median of 2.86 with a mean of 2.96 (SE = 0.0281), while the excluded participants showed a median of 3.00 with a mean of 2.97 (SE = 0.0295). A two-sided Mann–Whitney U test revealed no significant difference between the two groups. These results suggest that the exclusion of participants with unsmooth interaction flows is unlikely to have a substantial impact on the generalizability of the findings, given the scope of the data collected in this experiment.

### 4.2 Hypotheses testing

#### 4.2.1 Categorization of instruction abstractness

Participants were divided into two groups: those whose situation descriptions for the robot’s facial expression explicitly referred to emotions or physical conditions, and those whose descriptions did not. To determine whether there were emotions or physical conditions in the description, we used GPT-5 by OpenAI, Gemini 2.5 Pro by Google DeepMind, and Claude Opus 4.1 by Anthropic, and took a majority vote. Examples that were concrete and included emotions or physical conditions were “when you are happy after winning the lottery” or “when you feel pain after falling off a bicycle,” whereas examples of abstract instructions without such references were “when you are about to be late for school” or “when your lie is discovered.” Participants whose instructions in both trials did not include specific emotions or physical conditions were categorized as the abstract group (n = 200), while those who provided at least one instruction containing these references were categorized as the concrete group (n = 127).

The abstract group consisted of 80 males, 119 females, and 1 other, and the mean age was 45.9 years. The concrete group consisted of 52 males and 75 females, and the mean age was 49.2 years. In the analysis of personality traits, normalized scores were used for each of the Big Five factors to adjust for differences in the number of response scales between adults and children. The abstract group showed average scores of 0.577 (Extraversion), 0.662 (Agreeableness), 0.520 (Conscientiousness), 0.519 (Neuroticism), and 0.606 (Openness), whereas the concrete group showed averages of 0.581, 0.673, 0.505, 0.558, and 0.592, respectively. The two-sided Mann-Whitney U test did not show significant differences in all Big Five factors between the groups.

#### 4.2.2 Analysis results

**FIGURE 3:**
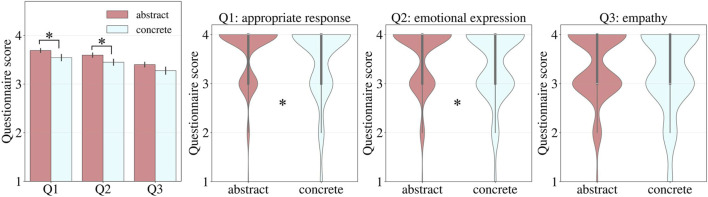
Analysis results of questionnaire responses based on how situations were specified (left: mean scores, right: violin plots, *p < 0.05).

The results of the one-sided Mann–Whitney U test comparing questionnaire evaluations between the abstract and concrete groups are shown in Figure 3. The median (mean ± SE) scores of the abstract group vs. the concrete group were as follows: Q1: 4.00 (3.69 ± 0.0391) vs. 4.00 (3.54 ± 0.0640), Q2: 4.00 (3.60 ± 0.0432) vs. 4.00 (3.45 ± 0.0620), Q3: 3.00 (3.40 ± 0.0476) vs. 3.00 (3.28 ± 0.0722). For Q1 (appropriate response) (U = 13819.0, p = 0.047) and Q2 (emotional expression) (U = 14090.0, p = 0.026), the abstract group, those who did not specify particular emotions or physical conditions, rated significantly higher evaluations. In contrast, no significant difference was observed for Q3 (empathy) (U = 13491.5, p = 0.147). The effect sizes based on Cliff’s delta were δ = 0.088 for Q1, δ = 0.109 for Q2, and δ = 0.062 for Q3. These relatively modest values could be influenced by the use of a 4-point Likert scale, which offers limited sensitivity for detecting subtle differences between groups. In summary, H1 and H2 were verified, but H3 was not verified.

### 4.3 General analyses

#### 4.3.1 Evaluation across facial expressions

**FIGURE 4:**
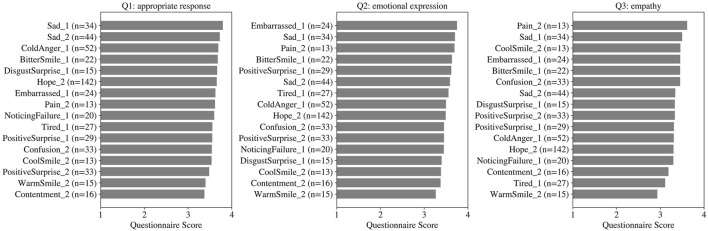
Mean questionnaire scores for each facial expression that appeared in at least 10 participants. The numbers following the expression names indicate intensity, with 1 representing low and 2 representing high.

The average questionnaire scores for each facial expression are presented in Figure 4. The analysis was limited to expressions that were performed to at least 10 participants. In the case of Q1 (appropriate response), the highest-rated expressions were Sad (low-intensity), Sad (high-intensity), and ColdAnger (low-intensity) in descending order. Regarding Q2 (emotional expression), the top-rated expressions were Embarrassed (low-intensity), Sad (low-intensity), and Pain (low-intensity). With respect to Q3 (empathy), the highest evaluations were given to Pain (high-intensity), Sad (low-intensity), and CoolSmile (high-intensity).

#### 4.3.2 Correlations between Big Five and questionnaire results

**FIGURE 5:**
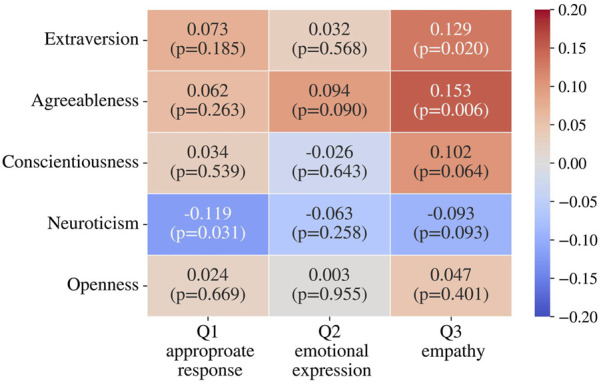
Correlation between participants’ personality traits and questionnaire results.

The correlations between the Big Five personality traits obtained before the experiment and the questionnaire responses were analyzed using Spearman’s rank correlation coefficient. The results are shown in Figure 5. In general, there were no strong correlations between the personality traits and the results of the questionnaire. Although statistically significant negative correlation between Q1 and neuroticism ( ρ = −0.119, p = 0.031) and positive correlations between Q3 and extraversion ( ρ = 0.129, p = 0.020) and agreeableness ( ρ = 0.153, p = 0.006), the observed correlations were very weak ( ρ< 0.2), suggesting limited practical impact.

## 5 Discussion

### 5.1 Emotion attribution to the robot

Regarding the Agency-related items (Q1 and Q2), the abstract group, in which participants did not explicitly specify particular emotions or physical conditions, scored significantly higher evaluations. Within the theoretical framework of human-robot interaction, it is argued that agency is determined by interactivity, autonomy, and adaptability (Jackson and Williams, 2021). When no explicit emotions or physical conditions were specified, the robot’s behavior was more likely to be interpreted as if it had inferred the appropriate emotion and facial expression, not as merely acting according to given instructions. This may enhance the perceived autonomy and adaptability of the robot. In addition, the absence of predefined emotional labels broadened participants’ interpretive latitude, expanding the range of expressions considered appropriate, resulting in an effect on the participants’ perception of the robot’s adaptability. In summary, the perceived agency was increased when the robot’s expressive behavior was generated by the abstract situational instructions without emotions or physical conditions.

These findings can also be explained from the perspective of predictive processing (Clark, 2013). Human perception arises from the integration of bottom-up sensory input and top-down predictions. In this experimental setting, bottom-up input can be understood as the perception of the robot’s facial expression together with the human’s instruction. Top-down prediction refers to inferences generated from internal models based on prior knowledge and expectations. When the instruction given to the robot is abstract and the bottom-up information is limited, people need to rely more heavily on top-down predictions to compensate for the missing information. During this compensation process, assumptions about internal states of the robot, such as the reason why it chooses this expression, are likely to be invoked. Therefore, the robot’s agency is perceived more strongly.

However, with regard to the Experience-related items (Q3), no significant differences were observed. As mentioned earlier, the absence of specific emotional instructions may have broadened participants’ interpretive latitude, leading to cases where the robot’s expression did not necessarily align with the participant’s own feelings. In such cases, participants may have perceived the expression as reasonable for someone else, even if it did not match their personal experience. In addition, the experimental setting may affect this result. In this study, the robot only performed the participants’ experiences, rather than its own experiences. This lack of perceived first-person experience of the robot could have weakened the impression of empathy. In studies on human interaction, it has been shown that when people mention having similar experiences, it can increase the sense of empathy perceived by others (Hodges et al., 2010). For future study, it may be beneficial to design scenarios in which, when responding to participant instructions, the robot explicitly refers to its own similar past experiences. (e.g., “I’ve been through a similar situation, and this was how I felt.”) Such contextual framing may enhance the perception of empathy between the robot and the participant.

It is also important to acknowledge methodological limitations. For instance, the empathy was assessed using a single-item Likert question, rather than a multi-item scale, which might not be sensitive enough to detect more subtle feelings of empathy. In addition, other factors such as the interaction duration and the robot’s appearance may also have influenced participants’ perceptions. Particularly, the robot was designed to appear as a junior high school-aged child. This may have impacted perceived empathy, especially in scenarios that are atypical for children of that age, for example, receiving congratulations at a wedding.

### 5.2 Trends across facial expressions

In general, most expressions achieved average ratings above 3 on a 4-point scale across all questionnaire items. This result indicates that the robot was largely successful in presenting expressions that participants regarded as appropriate to the given situations, and that the repertoire of expressions was sufficiently broad to address many different contexts. Although the repertoire covered a wide range of contexts, some situations mentioned by participants appeared to fall outside of its scope, such as “when you eat sour pickled plum,” and “when you are desperately fighting an enemy.” Additional categories of expression could further improve emotional interaction in future work.

Compared to positive expressions (e.g., Positive Surprise, Hope), negative expressions (e.g., Sad, Embarrassed, Pain) tended to receive higher ratings across all three evaluation items. This aligns with previous research on human-human interaction, which has reported that sharing negative episodes enhances empathy more than sharing neutral episodes, even though the experimental conditions differ (Cheng et al., 2024). In addition, regarding pain, which received the highest ratings for Q3 (empathy), previous studies have shown that observing facial expressions of pain can trigger empathic responses in the observer (Xiong et al., 2019). It is suggested that participants may have interpreted the robot’s negative facial expressions not as mere reactions, but as emotional resonance, and recalled their past experiences and emotions again. As a result, they were more likely to feel that the robot empathized with them.

### 5.3 Relationship with Big Five personality traits

All absolute correlation coefficients were below 0.2, suggesting that there is little meaningful relationship between personality traits and the questionnaire scores. This suggests that participants’ personality traits did not strongly influence the evaluation of the impressions towards the robot in this experiment. Although previous studies have reported that personality traits influence human-robot interaction (Kabacin̈ska et al., 2025), within the scope of this experiment, impression evaluations are considered to be carried out without being strongly affected by personality traits.

### 5.4 Recovery from errors

In this experiment, after the robot performed a facial expression, participants were asked how they felt about it, and if they responded negatively, the robot attempted to present an alternative expression. While some participants were satisfied after the first retry, there was a general trend indicating that the occurrence of retries led to lower evaluation scores in the questionnaire. In human-robot interaction, recovery strategies following failures have shown limited success in restoring trust, while apologies and explanations are considered relatively effective (Esterwood and Robert, 2025). In this experiment, the robot’s responses were generated using the LLM, and therefore, the robot did not consistently provide apologies or explanations when retrying facial expressions.

In some cases, participants provided specific requests during retries, such as asking the robot to adjust certain parts of the face. However, the current system was designed to regenerate entire facial expressions at the level of emotion labels and did not support the changes of facial action units or actuator movements. This factor prevented the robot from following such detailed user requests. For future development, these issues should be taken into account to improve the system.

### 5.5 Limitation

Since the experiment was conducted as part of an exhibit at the Expo 2025, it was necessary to minimize the burden on participants when completing the post-experiment questionnaire. As a result, the evaluation of the robot was limited to only three questions, and detailed assessments could not be conducted. Future studies should incorporate more comprehensive measures, such as the Dimensions of Mind Perception questionnaire (Gray et al., 2007) or the Perceived Agency Scale (Trafton et al., 2024), to allow for more detailed evaluation.

The android robot used in this study had the appearance of a junior high school-aged child. Therefore, many of the participants perceived the robot as younger than themselves. The use of a robot with a different age or gender could lead to different results.

In addition, some of the facial expressions used in this study were interpreted inconsistently as described in Section 3.4. This suggests that some facial expressions may not be universally intuitive, highlighting potential limitations in their generalizability. Further study is needed on this issue. However, in the process of this study, when participants thought that the expression performed by the robot was not good, they could respond negatively, and such instances were excluded from the evaluation as an unsmooth interaction flow. Therefore, we consider that the validations of each facial expression had no significant impact on the experimental results.

## 6 Conclusion

In this study, we implemented a total of 63 facial expressions on the android robot Nikola, including not only basic emotions but also complex emotions, physical conditions, and variations in emotional intensity. We conducted a large-scale experiment at Expo 2025 Osaka, Kansai, Japan. Participants provided verbal descriptions of various situations, to which the robot responded by performing a corresponding facial expression. The robot’s responses were assessed along three key dimensions: appropriateness, emotional expression, and empathy. The results showed that most of the expressions received high ratings, with average scores of 3 or higher on a 4-point scale, indicating that the prepared repertoire of expressions was generally perceived as appropriate across a wide range of situations.

In addition, when participants described situations in more abstract terms, without explicitly stating specific emotions or physical conditions, the robot was perceived to have higher agency, including the ability to respond appropriately and express emotions. It is suggested that the absence of explicit emotional cues may have enhanced the sense of the robot’s autonomy and adaptability in inferring the emotion and encouraged participants to interpret the expression in more diverse ways. On the other hand, there was no significant difference in perceived empathy based on the level of abstraction in the instructions with this experimental setting.

This study provides new insights into the field of human–robot interaction by demonstrating how people perceive robots that flexibly express emotions based on contextual input, and showing that the abstractness of user instructions influences emotional attribution. While this experiment focused on the abstractness of human instructions, the gained insights may have broader applications in designing robot behaviors, such as when a robot shares its own experiences along with facial expressions. Further study would be required to explore the applicability of these findings in such contexts. In future work, we would like to expand the range of expressions to accommodate more diverse situations, improve interaction system design, and conduct more detailed questionnaire evaluations.

## Data Availability

The datasets presented in this article are not readily available because of ethic restrictions. Requests to access the datasets should be directed to AF, ayaka.fujii.wu@riken.jp.
