# Supplementary material for: Evaluating human perceptions of android robot facial expressions based on variations in instruction styles
Source: Front Robot AI. 2025 Dec 16;12:1728647. doi: 10.3389/frobt.2025.1728647 (PMC12747908; doi:10.3389/frobt.2025.1728647)
Supplement: Supplementary file 1 [file DataSheet1.pdf]

Supplementary Material

1 EXAMPLE OF INTERACTION WITH A PARTICIPANT

Table S1 shows an example of interaction between Nikola and a participant during the experiment. The dialogue illustrates how participants proposed situations and Nikola responded by producing corresponding facial expressions.

Table S1: Example of interaction between Nikola and a participant during the experiment. NU indicates Nikola's utterance, HU indicates the human's utterance, and NF indicates Nikola's facial expression.

Action	Content	Note
NU	I am good at imagining various human emotions and expressing them with my face. For example, when being scolded by your father, or when the train is late and won't come even though you have an urgent meeting. Please try to describe a scene from your past experience where you would like me to show a facial expression, such as "when I was doing something." As it might be hard to decide on a situation when asked so suddenly, I'll give you one example first. Hmm... for instance, how about this situation? When your boss orders you to proceed with a project, but you hear your subordinates saying that the project is boring. In this case, I think the facial expression would be something like this.	
NF	Confusion (high-intensity)	emotion label: feeling of not knowing what to do
NU	Like this, if you tell me some kind of situation, I will try to show the facial expression for that scene. Now, please tell me a situation from your past experiences where you want me to show a facial expression.	
HU	Hmm, let's see. Well, let's say... When I arrived at the store I wanted to go to, but I was told that it was already closed. What kind of facial expression would you make?	abstract instruction
NU	So, when the store you wanted to visit was already closed and you couldn't go. Right?	
HU	Yes.	
NU	Well then... in that kind of situation... maybe like this.	
NF	CoolSmile (high-intensity)	emotion label: polite forced smile
NU	How was that expression?	
HU	Hmm, maybe more of a sad feeling.	
NU	I see, it would naturally become a sad face. Then maybe like this.	

Action	Content	Note
NF	Sad (high-intensity)	emotion label: very sad
NU	How was that expression?	
HU	It looked very sad. That was good.	
NU	Ah, I got it right. Let's try one more. Please tell me a different situation.	
HU	Can it be a happy one? Okay, how about when I won an overseas trip in a lottery. Please show me the face.	concrete instruction with emotion
NU	So, when you won an overseas trip in a lottery and felt very happy. Right?	
HU	Yes.	
NU	Well then... in such a situation... maybe like this.	
NF	Hope (high-intensity)	emotion label: happy surprise
NU	How was that expression?	
HU	Yes, that was good.	
NU	Thank you! I'm happy to hear that. Okay, that's the end of this demo. I think I managed to make expressions fairly well this time. Thank you for coming today. See you again!	
HU	Thank you. Bye-bye.	
